# Preparation, Characterization, and Evaluation of Enzyme Co-Modified Fish Gelatin-Based Antibacterial Derivatives

**DOI:** 10.3390/polym16070895

**Published:** 2024-03-25

**Authors:** Mingyao Zhu, Jing Xiao, Yaru Lv, Xin Li, Yangyi Zhou, Miaomiao Liu, Chunxiao Wang

**Affiliations:** 1 State Key Laboratory of Biobased Material and Green Papermaking, Qilu University of Technology, Shandong Academy of Sciences, Jinan 250353, China; zhumingyao1999@163.com (M.Z.); lvyaru230@163.com (Y.L.); lixin201003@163.com (X.L.); z1347917482@163.com (Y.Z.); 2 Shandong Loncote Enzymes Co., Ltd., Linyi 276000, China; liumiaomiao@longda-enzyme.com (M.L.); wangchunxiao@longda-enzyme.com (C.W.)

**Keywords:** fish gelatin, protease, glutamine aminotransferase, derivative, glucose oxidase

## Abstract

Fish gelatin (FG)-based wound dressings exhibit superior water absorption capacity, thermal stability, and gelation properties, which enhance the performance of these dressings. In this study, our objective was to investigate the conditions underlying the enzymatic hydrolysis of FG and subsequent cross-linking to prepare high-performance gels. A two-step enzymatic method of protease-catalyzed hydrolysis followed by glutamine transglutaminase (TGase)-catalyzed cross-linking was used to prepare novel high-performance fish gelatin derivatives with more stable dispersion characteristics than those of natural gelatin derivatives. Compared with conventional TGase cross-linked derivatives, the novel derivatives were characterized by an average pore size of 150 μm and increased water solubility (423.06% to 915.55%), water retention (by 3.6-fold to 43.89%), thermal stability (from 313 °C to 323 °C), and water vapor transmission rate, which reached 486.72 g·m^−2^·24 h^−1^. In addition, loading glucose oxidase onto the fish gelatin derivatives increased their antibacterial efficacy to >99% against *Escherichia coli* and *Staphylococcus aureus*.

## 1. Introduction

At present, the demand for dressings in the wound healing and skin care industries is high [1,2]. Natural polymers commonly used in the manufacture of wound dressings mainly include chitosan, cellulose, gelatin, alginate, hyaluronic acid, chitin, etc. [3,4]. Gelatin is the denaturation product of collagen under certain conditions, and it is an inhomogeneous protein mixture. Among the 18 amino acids that make up gelatin protein, 7 are necessary for the human body, and it does not contain cholesterol, which is an ideal protein source. Fish gelatin (FG) dressing is a type of biological dressing that has the advantages of low antigenicity, high biodegradability, and no cytotoxicity, which enhances wound healing [5,6,7]. Fish gelatin is widely used in wound [8,9] and hemostatic dressings [10]. In addition, fish gelatin and mammalian gelatin have similar functional properties, while fish gelatin avoids the health and socio-cultural issues associated with the use of mammalian-sourced gelatin [11] and is considered as a potential alternative to mammalian gelatin with a wide range of applications [12].

Ideal dressings should show good dispersion properties, swelling, water retention, and water vapor permeability [13]. Haririan et al. [14] prepared a gelatin dressing with a swelling rate and water vapor permeability of up to 657.9% and 8.6 × 10^−9^ g/m·h, respectively. Zhang et al. [15] prepared a BSP–gelatin hydrogel dressing with a swelling rate of up to 45 ± 2.6%, and Ding et al. [16] prepared a gelatin-based immunomodulatory hydrogel dressing with a swelling rate of approximately 400% and an average pore size of 80 μm. Compared to mammalian gelatin, FG shows lower levels of proline and hydroxyproline, lower gelation capacity, and limited water absorption and retention capabilities [17,18]. Cross-linking, a method that can effectively improve these properties [19], comprises three types: physical, chemical, and biological (enzyme) cross-linking. Physical modifications have limitations in improving performance and may create stability issues over time [20], while chemical modifications produce biotoxic byproducts and are not widely used in dressings [21]. Enzyme cross-linking is greener, safer, and non-toxic compared with the other two methods and has broader application prospects. Glutamine aminotransferase (TGase) is an acyltransferase that can catalyze the cross-linking reaction between the γ-carboxyamide group in polypeptide chain glutamine residue and the primary amine group in various amine organics. Proteins can be modified to change their functional properties, and they are widely used in the modification of gelatin because of its high-cost performance [22]. Long et al. [23] used TGase as a cross-linking agent to prepare gelatin dressings with uniform pore sizes (100 μm) and porosity (53.51±3.45%). Cheng et al. [24] prepared TGase cross-linked gelatin hydrogel dressings with a swelling rate of 400%.

In addition to improving gel properties, some enzymes can impart antibacterial properties to gel dressings. For example, glucose oxidase (GOD) is an enzyme that can produce H_2_O_2_, while hydrogen peroxide is a commonly used disinfectant in mammals. H_2_O_2_ has poor stability and is easy to decompose, and the -OH generated by its oxidative decomposition has strong oxidation, which can inhibit and kill pathogenic microorganisms. During the bacterial infection of a wound, glucose at the wound site can act as a nutrient for the bacteria to promote their growth, and reducing the glucose concentration at the wound infection site can inhibit the growth of the bacteria, while GOD can catalyze the glucose reaction; on the one hand, it can inhibit the growth of the bacteria through starvation, and on the other hand, the catalytic production of H_2_O_2_ can play an antibacterial role. Fang et al. [25] prepared thermosensitive hydrogels loaded with glucose oxidase (GOD), which completely inhibited the growth of *Staphylococcus aureus* and *Escherichia coli* and promoted wound healing, in addition to being highly biocompatible. Division et al. [26] prepared a GOD–peroxidase-conjugated alginate diamine PEG-g-poly (PEGMA) dry gel that released 57% GOD-loaded and 63% peroxidase-loaded dry gel after 24 h, resulting in a significant improvement in wound closures, demonstrating its potential for treating diabetic wounds. However, the use of GOD for treating wound infections has not been widely explored owing to its lack of stability in the wound microenvironment.

According to the above research background, it is known that fish gelatin is a potential derivative material with wide application prospects, but the gelation and water absorption and retention properties of fish gelatin are limited, so it needs to be modified, in which enzymatic modification is widely used with green, safe, and non-toxic characteristics. In this study, we proposed to use protease to hydrolyze gelatin and then cross-link it with TG enzymes, which can regulate the physicochemical properties and application performance of gelatin in a wider range according to the parameter requirements of different derivatives and application fields, and TG enzymes are able to cross-link glucose oxidase to gelatin protein, which improves the bacteriostatic properties of the derivatives.

## 2. Materials and Methods

### 2.1. Materials and Equipment

Balsa FG M03 (powder) was purchased from Jinan, Shandong, China, and TGase enzyme (7800 U/g), alkaline protease (500,000 U/g), neutral protease (200,000 U/g), keratinase (130,000 U/g), glucose oxidase (10,000 U/g), and food-grade enzyme preparations were obtained from Loncote Enzyme Preparation Co, Linyi, Shandong, China. *Escherichia coli* (BL21) and *Staphylococcus aureus* (ATCC25923) were deposited in the Industrial Microbial Strain Deposit and Management Center, Beijing, China.

Phosphate-buffered saline (PBS) buffer was used for gelatin preparation. A test solution (Test Solution A) was created by dissolving 8.298 g of NaCl and 0.368 g of calcium chloride dihydrate in 1 L of deionized water. The ionic content of this solution was equivalent to that of human serum or traumatic exudates and was used for dispersion characterization experiments.

Equipment: Fourier-transform infrared spectrometer, model Nicolet10, Thermo Fisher Scientific (Waltham, MA, USA); thermo-gravimetric analyzer, model TGA 2 (SF), METTLER TOLEDO; X-ray diffractometer, model D8-ADVANCE, Bruker AXS; scanning electron microscope, model S-4800, Hitachi, Tokyo, Japan; double single-side purification worktable, model SW-CJ-2FD, Suzhou Purification Equipment Co. (Suzhou, China).

### 2.2. Preparation of Fish Gelatin (FG) Derivative

To prepare the gelatin self-gel, M03 gelatin was dissolved in PBS solution to create a 6.67% gelatin solution (pH 7.0) that was cooled at 4 °C for 12 h to obtain gelatin self-gel. The gelatin self-gel was pre-frozen at –20 °C for 48 h after vacuum freeze-drying for 48 h to obtain a gelatin self-gel derivative.

To prepare the TGase cross-linking gel, M03 gelatin was dissolved in PBS solution to create a 6.67% gelatin solution (pH 7.0). The gelatin solution was treated with 100 u/mL of TGase which was added to initiate cross-linking at 40 °C for 1 h, and then, it was inactivated at 80 °C to obtain the TGase cross-linking gel. The cross-linking gel was pre-frozen at –20 °C for 48 h and then vacuum freeze-dried for 48 h to obtain the TGase gelatin derivative.

To prepare the dual-enzyme gel, M03 gelatin was dissolved in PBS solution to create a 6.67% gelatin solution (pH 7.0). This solution was treated with a final protease concentration of 20 u/mL, hydrolyzed for 2–15 min at 50 °C, and then inactivated in a boiling water bath for 10 min to obtain gelatin hydrolysate. Then, 100 u/mL of TGase was added to the gelatin hydrolysate at 40 °C, cross-linked for 1 h, and inactivated at 80 °C to obtain a dual-enzyme gelatin derivative. This derivative was pre-frozen at –20 °C for 48 h and then vacuum freeze-dried for 48 h to obtain a dual-enzyme gelatin derivative. The gelatin hydrolysate was obtained by adding 100 u/mL of TGase to the gelatin hydrolysate to initiate cross-linking for 1 h, and then inactivated at 80 °C. The gelatin derivative was obtained by pre-freezing at –20 °C for 48 h and then freeze-drying at –20 °C for 48 h.

### 2.3. Dispersion Characteristic Experiment

A gel sample of a certain mass was weighed and placed in a suitable container with a plug. Test solution A was then added to the container at eight times the mass volume (*v*/*w*) of the gel sample. The gel was oscillated and the structure was observed at room temperature for 2 min and then allowed to stand for 2 h. If the gel fibers separated and no longer retained the original structure, the gel was considered dispersed.

### 2.4. Swelling Rate Experiment

The swelling rate experiment was based on the experimental method described by Taheri et al. [27]. Gelatin self-gels and enzyme gel samples were prepared. The gel samples were cut into 1 cm^3^ pieces and pre-frozen at –20 °C for 6 h, and then freeze-dried for 24 h with a vacuum freeze dryer. A freeze-dried gel sample was selected, its initial mass (*W*_0_) was recorded, and then, it was immersed in deionized water and placed on a shaking table at 60 rpm for 24 h. The sample was removed every 2 h, and its mass after absorbing surface moisture (*W_e_*) was accurately weighed and recorded. Three parallel controls were set for each group of samples, and the swelling rate (*SR*) was calculated as follows:

$$SR\left(\%\right)=\frac{W_{e}-W_{0}}{W_{0}}\times 100\%$$

### 2.5. Water Retention Test

The freeze-dried gel was weighed and recorded as *W*_0_. The gelatin and enzyme gel were removed at room temperature, incubated in deionized water for 2 h, and then weighed as *W_T_*. The gel was maintained at room temperature for 10 h and weighed every 2 h. Three parallel samples were tested for each gel.

$$Water\;retention\;rate\left(\%\right)=\frac{W_{t}-W_{0}}{W_{T}-W_{0}}\times 100\%$$

### 2.6. Water Vapor Transmittance (WVP) Test

The *WVP* test was performed at room temperature (20–25 °C). A glass vial was filled with 10 mL of deionized water and the derivative was turned into a disc form. The mouth of the glass vial was covered with a sample inside and then sealed. The glass vial was then covered with the derivative and placed in a dryer containing silica gel. The temperature inside the dryer was maintained at 37 °C for 24 h. The glass vial was accurately weighed before and after drying. The experiment was repeated thrice. *WVP* (g·m^−2^·24 h^−1^) was calculated as follows:

$$WVP=(W_{1}-W_{2})\times 1000\times 24/T$$

where *W*_1_ and *W*_2_ represents the mass (g) of the container, sample, and liquid before and after the test (g), and *T* is the test time (h).

### 2.7. Molecular Weight Analysis of Fish Gelatin Hydrolysates

A 1 mg/mL sample solution was prepared, and after complete dissolution, 80 μL of the sample solution was taken and mixed well with 20 μL of protein upstaining solution, boiled for 5 min, and centrifuged, and 10 μL of upstaining solution was taken and run at 60 V. The gel was electrophoresed with a Coomassie stainer. The gel after electrophoresis was stained with Coomassie and scanned and photographed with a gel imager for analysis.

### 2.8. Fourier-Transform Infrared Spectroscopy (FTIR) Test

Approximately 2 mg of freeze-dried sample and 200 mg of KBr were placed in a mortar, ground evenly, pressed into transparent slices, and scanned using a Fourier-transform infrared spectrometer in the range of 4000–500 cm^−1^.

### 2.9. X-ray Diffraction (XRD) Test

The freeze-dried sample was ground into a powder and analyzed using an X-ray diffractometer with a copper target. The scanning range was 5–80°, the scanning speed was 30°/min, and the step length was 0.02.

### 2.10. Thermogravimetric (TGA) Test

Samples were dried in a vacuum at 60 °C for 48 h, and 0.1 g of each sample was weighed and completely ground down using a grinder. An appropriate amount was extracted with tweezers and carefully placed in a crucible, gently compacted, and placed in a TGA tester, at a temperature range of 30–800 °C and heating rate of 10 °C/min.

### 2.11. Scanning Electron Microscopy (SEM) Analysis

Gel samples were prepared and freeze-dried using a vacuum freeze dryer. Freeze-dried samples were placed on an SEM platform loaded with a conductive adhesive and sprayed with a gold coating. The micromorphology of the gel was then observed using a scanning electron microscope, and the pore size was calculated using Nano Measurer software (1.2.5).

### 2.12. Determination of In Vitro Coagulation Index with BCI

The blood clotting index (BCI) was used to evaluate the in vitro coagulability of hemostatic materials. A sample was cut to 0.5 × 0.5 × 0.5 cm^3^ and placed into a 100 mL beaker, and the beaker was placed into a water bath at 37 °C for 5 min. Subsequently, 0.1 mL of mouse anticoagulant blood was gently added to the sample, and then, 0.02 mL of 0.2 M CaCl_2_ solution was added. After 5 min, 25 mL of deionized water was added to the beaker, and the solution was shaken at 37 °C at 50 rpm for 5 min. The solution was removed and the antibodies were measured at 545 nm with an ultraviolet spectrophotometer. For the control, 0.1 mL of mouse anticoagulant blood was added to a beaker with 25 mL of deionized water. The antibodies were measured at the same wavelength, which was assumed to be 100 as the reference value. The BCI was calculated as follows:

$$BCI=\frac{A_{Sample}}{A_{Blank}}\times 100$$

The smaller the *BCI* value, the better the clotting effect of the corresponding material.

### 2.13. Preparation of Glucose Oxidase (GOD)-Loaded Gelatin Derivative

GOD solutions with different concentrations (5000, 10,000, 15,000, and 20,000 U/mL) were prepared with 100 U/mL of TGase. A sample of dual-enzyme gelatin derivative was placed in the solution for complete impregnation. The fully impregnated dual-enzyme gelatin derivative was pre-frozen for 48 h and then vacuum freeze-dried for a further 48 h to obtain a bacteriostatic derivative loaded with GOD. The derivative sample was ground into powder and used to determine the bacteriostatic rate.

### 2.14. Bacteriostatic Experiments

After 12 h of culture, the bacteria were diluted with LB medium to obtain a bacterial suspension of approximately 1.0 × 10^6^ CFU/mL. Subsequently, 100 µL of bacterial suspension was evenly coated on a solid medium that was prepared in advance. The antibacterial gelatin derivative was allowed to fully absorb 10 mg/mL of glucose solution and was then placed on the coated medium and cultured at 37 °C for 12 h. The bacteriostatic zone was then observed and photographed.

To determine the inhibition rate, the bacteria were cultured for 12 h and diluted with LB medium to obtain a bacterial suspension of approximately 1.0 × 10^4^ CFU/mL to 9.0 × 10^4^ CFU/mL for backup; 50 mg of sample powder and 5 mL of deionized water were added to a test tube, and then, a glucose solution was added to obtain a final concentration of 10 mg/mL. The test tube was placed in a water bath at 37 °C for 5 min, and then, 0.1 mL of bacterial suspension was added, quickly mixed, and timed. For the positive control, LB liquid medium was used instead of the sample solution to conduct parallel experiments. The mixed solution was maintained at 37 °C for 20 min and subsequently turned into a solid plate using a pouring method and incubated at 37 °C for 24 h for colony counting.

The bacteriostasis rate calculation was as follows:

$$X=\frac{A_{0}-A_{1}}{A_{0}}$$

where *X* is the antibacterial rate (%), *A*_0_ is the number of recovered bacteria in the positive control group (CFU/mL), and *A*_1_ is the number of bacteria recovered in the experimental group (CFU/mL).

The bacteriostatic gelatin derivative prepared in Section 2.12 was fully absorbed by the 10 mg/mL glucose solution, and the bacteriostatic zone (zone of inhibition) and rate tests were performed after allowing the gelatin derivative to stand for 1, 2, 3, 4, 5, and 6 h.

## 3. Results and Discussion

### 3.1. Screening of Hydrolyzed Gelatin Proteases

**Figure 1:**
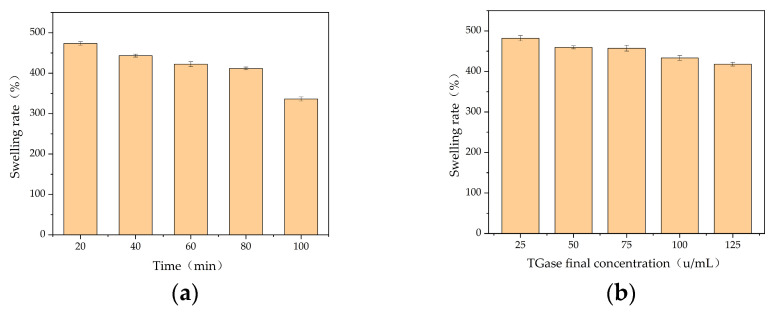
Dissolution rate of TGase cross-linked gels. Swelling rate with (**a**) TGase at different cross-linking times and (**b**) TGase at different final concentrations.

Only the 6.67% gelatin solution at 4 °C exists as static gelatin autogel, which is unstable at room temperature and completely melts in 5–6 min. Thus, gelatin autogel cannot be used as a gel dressing and must be modified. TGase is a cross-linking enzyme that can modify gelatin via cross-linking. An evaluation index of swelling rate, using the swelling rates associated with TGase-cross-linked gel at a TGase concentration of 100 u/mL, indicated that the swelling rate was negatively correlated with cross-linking time, with the highest swelling rate being 473.69% for a cross-linking time of 20 min. When the cross-linking time was 60 min, the swelling rate of the gel was negatively correlated with the amount of TGase added, with the highest swelling rate being 482.05% when the final concentration of TGase was 25 u/mL (Figure 1).

Our experimental results showed that the modification effect exerted on gelatin by TGase was limited and that new modification methods must be developed. In this experiment, a dual-enzyme gel was prepared by hydrolyzation with protease followed by cross-linking with TGase. Alkaline protease, neutral protease, and keratinase were selected as hydrolyzing proteases, with the final concentration of each being 20 U/mL. The results indicated that, among the three proteases, only the alkaline and neutral proteases hydrolyzed gelatin over a specific period, and TGase was able to cross-link gelatin that had been previously hydrolyzed by alkaline and neutral proteases for 10 min and 5 min, respectively, and return it to its original gelatin form.

**Figure 2:**
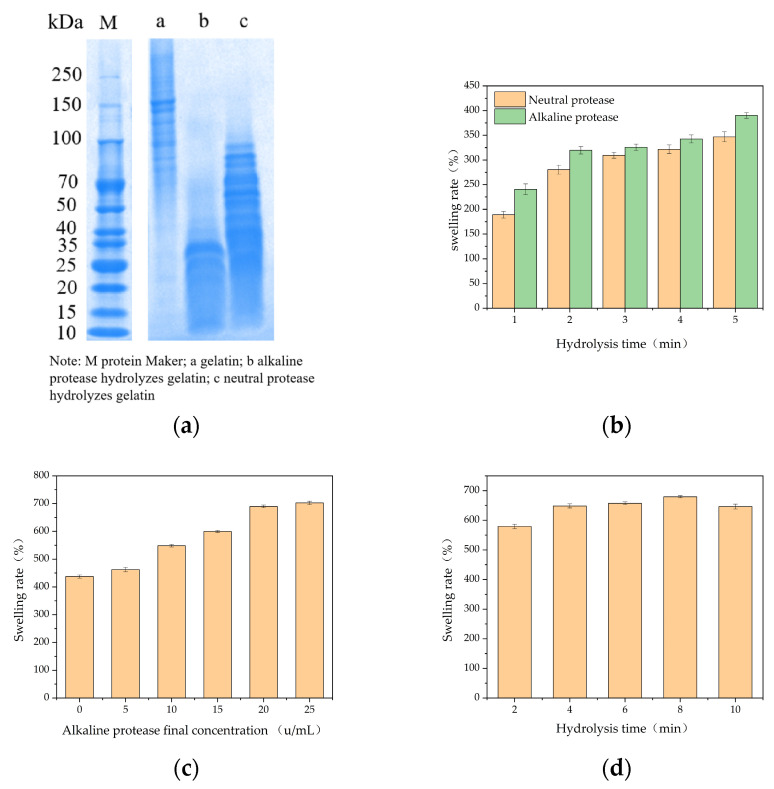
Dissolution rate of gels by dual-enzyme method. (**a**) Molecular weight analysis; (**b**) hydrolysis by different proteases; (**c**) different final concentrations of alkaline protease; (**d**) different hydrolysis times.

The molecular weight of fish gelatin after hydrolysis by alkaline protease was mainly concentrated below 35 kDa, and after hydrolysis by neutral protease, it was mainly concentrated below 100 kDa (Figure 2a). The swelling rates of the double-enzyme gels, prepared by hydrolyzing gelatin using the two proteases at different times, were compared (Figure 2a). The swelling rates of the gels increased with hydrolysis time within a range of 5 min. The swelling rates of the double-enzyme gels hydrolyzed by alkaline protease were better than those hydrolyzed by neutral protease. The swelling rate of the dual-enzyme gel varied with changes in the final concentration of alkaline protease and hydrolysis time (Figure 2b,c), indicating that it would be possible to obtain dual-enzyme gels with different swelling rates by altering the conditions of the enzyme environment. Thus, we selected the alkaline protease as the hydrolyzing enzyme for subsequent experiments.

### 3.2. Physical Properties of Dual-Enzymatic Gels

**Figure 3:**
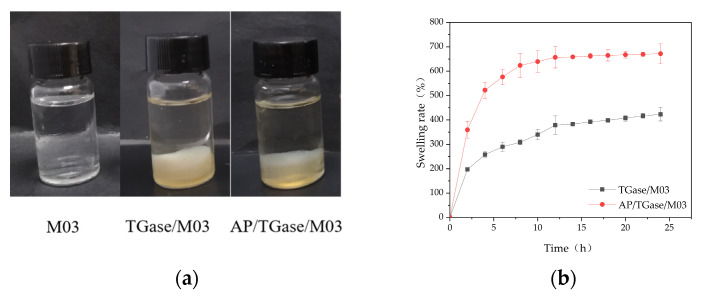

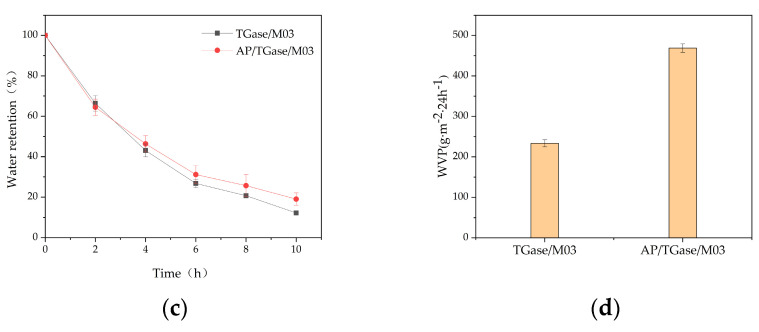
The (**a**) dispersion property; (**b**) solubility; (**c**) water retention; (**d**) water vapor permeability analysis of the cross-linked gelatin derivatives (TGase/M03 and AP/TGase/M03).

Good dispersion properties, swelling rates, water retention, and water vapor transmission rates are important for contact wound dressings. The dispersion property indicates the performance of dressings in terms of its ease of removal from wounds with exudates [28]. The M03 gelatin self-gel dissolved after sufficient shaking and standing, whereas the TGase/M03 gelatin gel and the AP/TGase/M03 gelatin gel maintained their original morphology without fiber dissolution or dispersion. This type of gel, when applied to wound dressings, maintains stability over time in the presence of wound exudate and provides continuous protection to the wound, improving its application value (Figure 3a).

Our solubility analysis indicated that both the TGase-cross-linked gel and the dual-enzyme gel showed rapidly absorbed liquid for 2 h and sustained their liquid absorption abilities for 12 h; subsequently, the rate of liquid absorption gradually slowed down until a solubility equilibrium was reached and maintained for 15 h (Figure 3b). The solubilities of the TGase-cross-linked gel and dual-enzyme gel were high. The dissolution rate of the dual-enzyme method gel increased from 423.06% to 671.66% compared with that of the TGase enzyme-cross-linked gel. The swelling rate determines the diffusion rate of nutrients and the accumulation of wound exudates [29]. The water retention performance curves of the TGase enzyme-cross-linked and the dual-enzyme gels show that the water retention rate of the TGase enzyme-cross-linked gel was 12.16% after 10 h at room temperature, whereas that of the dual-enzyme gel was 19% (Figure 3c). Good water retention provides a moist local environment, which is favorable for wound healing [30]. A suitable degree of water vapor permeability (WVP) prevents wound drying and moisture loss, maintains breathability, and reduces the risk of wound infection [31]. The water vapor transmission rate of the dual-enzyme gel improved more than the TGase-cross-linked gel, from 233.28 g·m^−2^·24 h^−1^ to 468.80 g·m^−2^·24 h^−1^ (Figure 3d).

The M03 FG autogel is soluble in aqueous solutions, which limits its application. The physical properties of gelatin prepared using hydrolysis followed by cross-linking were significantly better than those of gelatin prepared via direct enzyme cross-linking, indicating that the hydrolysis of gelatin into small-molecule polypeptides followed by re-cross-linking and reorganizing may help adjust its physical properties, such as solubility, water retention, and air permeability.

### 3.3. Optimization of the Preparation Process of Gelatin Derivative Using the Dual-Enzyme Method

Alkaline proteases, such as hydrolase and TGase, were used as hydrolyzing and cross-linking enzymes to prepare double-enzyme gelatin derivatives and to optimize the preparation process. Orthogonal tests were designed to optimize the conditions required for hydrolysis with alkaline proteases and cross-linking by TGase.

The optimal process for preparing gelatin derivatives was the dual-enzyme method (Table 1 and Table 2), which was created as follows: a gelatin concentration of 6%, a final alkaline protease concentration of 15 u/mL, a hydrolysis temperature of 50 °C, a hydrolysis time of 4 min, a pH of 8.0, a final TGase concentration of 100 u/mL, a cross-linking temperature of 45 °C, and a cross-linking time of 30 min. The above optimization process resulted in gelatin derivatives with a dissolution rate of 915.55%, a water vapor permeability of 486.72 g·m^−2^·24 h^−1^, a water retention rate of 43.89%, and a water vapor transmission rate of 486.72 g·m^−2^·24 h^−1^. The swelling rate of gelatin derivatives prepared by us was increased by 2–3 times compared to that in the study by Demir et al. [32] and more than 2 times compared to that in the study by Ching et al. [24]. The swelling rate and water vapor transmission rate of other biological dressings are shown in Table 3.

### 3.4. Fourier-Transform Infrared Spectroscopy (FTIR) Analysis

The self-gel (M03), TGase (TGase/M03), and dual-enzyme gelatin derivatives (AP/TGase/M03) were prepared separately; then, a sample of each gelatin derivative was ground into powder for characterization tests.

**Figure 4:**
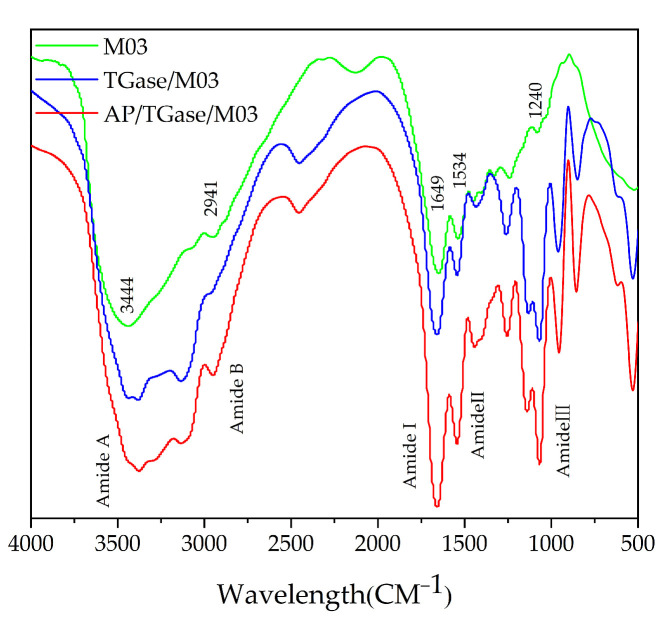
Fourier-transform infrared analysis pattern.

The FTIR spectra of the three materials, M03, TGase/M03, and AP/TGase/M03, are shown in Figure 4. The main bands of the FTIR spectra of M03 were 3444, 2941, 1649, 1534, and 1240 cm^−1^, and the intensities of the absorption peaks were significantly enhanced after hydrolysis, which may be attributed to the acquisition of higher NH_2_ and NH_3_^+^ contents during hydrolysis. The TGase/M03 and AP/TGase/M03 peaks were in similar positions, while the amide A band shifted from 3444 cm^−1^ to 3378 cm^−1^ and the amide I band shifted from 1649 cm^−1^ to 1662 cm^−1^ compared with that of M03, which may be due to the γ-carboxamide group of the glutamyl residue [39].

### 3.5. X-ray Diffraction (XRD) Analysis

**Figure 5:**
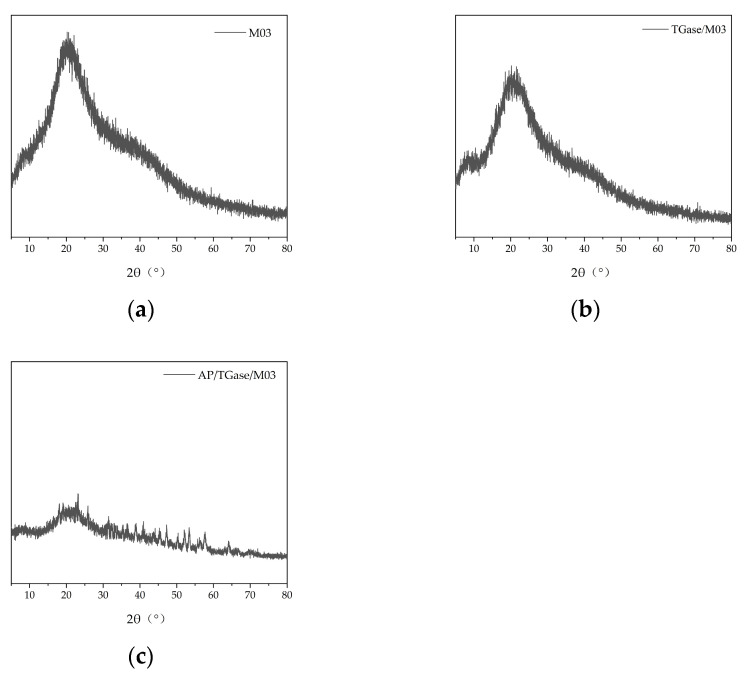
X-ray diffraction analysis diagrams. (**a**) M03 X-ray diffraction pattern; (**b**) TGase/M03 X-ray diffraction pattern; (**c**) AP/TGase/M03 X-ray diffraction pattern.

Figure 5 shows the XRD patterns of the three materials. The characteristic peaks of the M03 gel are flatter and the diffraction intensity is weaker in Figure 5b than in Figure 5a. This may be because the TGase-catalyzed cross-linking of gelatin changed the crystal structure. XRD analysis can characterize differences in the triple-helix structure of gelatin. Figure 5b shows that the addition of TGase significantly reduced the diffraction intensity of the characteristic peaks, implying that its triple-helix structure was reduced. The TGase-catalyzed cross-linking reaction typically forms ε-(γ-Glu)-Lys isopeptide bonds that hinder the formation of hydrogen bonds, leading to the reduction in the triple-helix structure [40]. Figure 5c shows that the crystal structure of gelatin had noticeably changed following alkaline protease-catalyzed hydrolysis, reducing the triple-helical structure.

### 3.6. Thermogravimetric (TGA) Analysis

**Figure 6:**
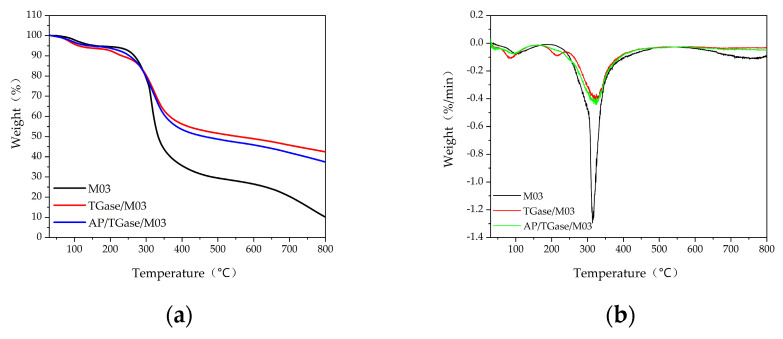
Thermogravimetric analysis plots. (**a**) TG plot; (**b**) TGA plot.

The three gelatin derivatives were analyzed by thermogravimetric analysis to evaluate their thermal stability, and the results are shown in Figure 6.

As shown in Figure 6, weight loss (%) can be divided into two stages according to the temperature: 60–120 °C, which is weight loss mainly due to the loss of free and bound water and the destruction of hydrogen bonds between protein chains [41], and 240–400 °C, which is a stage of rapid weight loss mainly due to the breakage of peptide bonds between the peptide chains of gelatin proteins and their further degradation into peptides and amino acids, followed by the destruction of amino acids via deamination and dehydration [42]. The thermal decomposition temperature of the M03 gelatin derivative was 313 °C, whereas that of the TGase/M03 and AP/TGase/M03 derivatives was 323 °C. A TGase-induced ε-(γ-Glu)-Lys isopeptide bond is approximately 20-fold stronger than a noncovalent bond, which may be a reason for the elevated thermal decomposition temperature [43]. The thermal decomposition temperature affects the triple-helix structure of gelatin proteins, which are relatively stable and more tightly bound to water at lower temperatures. Enzymatic cross-linking promotes the formation of a more stable protein network structure in the derivative and prevents the evaporative loss of water. Enzymatic cross-linking improves the thermal stability of FG, resulting in FG-based derivatives with better thermal properties.

### 3.7. Microscopic Morphological Analysis

**Figure 7:**
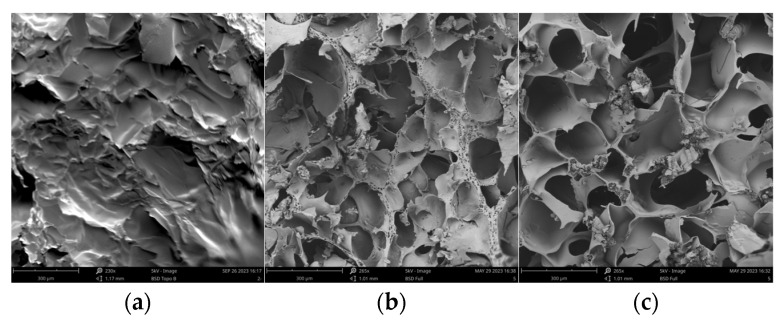
Microscopic morphology of gel derivatives. (**a**) M03; (**b**) TGase/M03; (**c**) AP/TGase/M03.

As shown in Figure 7, the cross section of the vacuum freeze-dried M03 autogel showed irregular scales with few pore structures. After cross-linking with TGase, the pore structures increased significantly and were more heterogeneous. Thus, hydrolysis and subsequent cross-linking significantly increased the regularity of the pore structures of gelatin, with an average pore size of 150 μm.

### 3.8. Analysis of Blood Coagulation In Vitro

**Figure 8:**
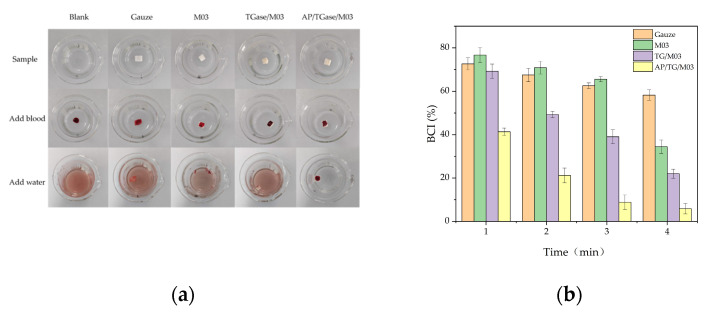
Coagulation of mouse blood when exposed to different derivatives. (**a**) An image of the coagulation process of different samples and (**b**) a coagulation index corresponding to the different samples.

The coagulation property of a dressing is important for its practical application. Coagulation refers to the process of blood changing from liquid to a coagula state through biochemical reactions. We tested the in vitro coagulation properties of different materials, as shown in Figure 8.

Figure 8a shows the coagulation of mouse blood after exposure to various derivatives. The blood was exposed to nothing (control/blank), gauze, the M03 gelatin derivative, the TG/M03 gelatin derivative, and the AP/TG/M03 gelatin derivative, and deionized water was added 5 min after contact with the material. The color depth of the non-coagulated part of the blood was observed. The coagulation effect of the AP/TG/M03 gelatin derivative was significantly different from the others. The blood in the other materials barely coagulated; the rinsed water turned red and the M03 gelatin derivative melted when water was added. We calculated the in vitro coagulation index (BCI) value of each sample to evaluate the hemostatic effect of the derivative, and the smaller the BCI value, the better the hemostatic effect of the material. Figure 8b shows the change in the BCI values of the different samples over time. The BCI value of the AP/TG/M03 gelatin derivative was the lowest; at 4 min of coagulation time, the BCI was only 5.846%, and the coagulation effect was significantly better than that of other materials. In addition, Xie et al. [44] prepared a sponge dressing with a BCI value of 21.9%, while our prepared gelatin derivative had a BCI value of 5%.

### 3.9. Gelatin Derivative Loaded with GOD

To enhance the performance of gel derivatives, a dual-enzyme gel was enzymatically modified to improve the antibacterial properties. Glucose oxidase (GOD) is an enzyme that produces H_2_O_2_, which consumes glucose and inhibits bacterial growth via starvation. We used TGase to cross-link and immobilize GOD in dual-enzymatic gelatin derivatives to produce bacteriostatic derivatives and subsequently tested the bacteriostatic properties.

**Figure 9:**
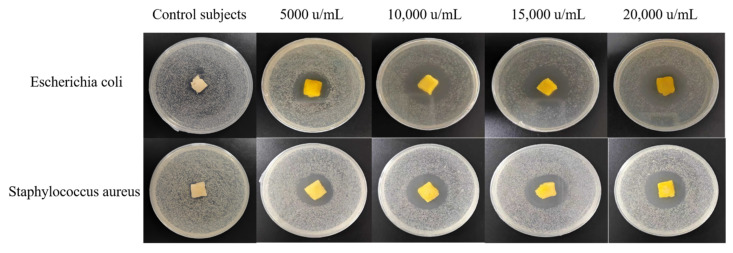
A photograph of the zones of inhibition of the antibacterial derivatives containing different amounts of glucose oxidase.

The antibacterial gelatin derivative showed clear zones of inhibition for both *E. coli* and *S. aureus*, which increased in diameter as the concentration of the GOD solution in the antibacterial gelatin derivative increased (Table 4 and Figure 9). The largest of zone of inhibition was observed at a GOD concentration of 20,000 u/mL, reaching 39.47 and 34.65 mm for *E. coli* and *S. aureus*, respectively. The inhibitory gelatin derivatives prepared using this method displayed a good inhibitory effect. The antibacterial gelatin derivatives with different concentrations of GOD were ground into powders to determine their inhibition rate and then changed to a solution to further evaluate the inhibitory properties of the gelatin derivatives via quantitative experiments. The results are shown in Table 5.

**Figure 10:**
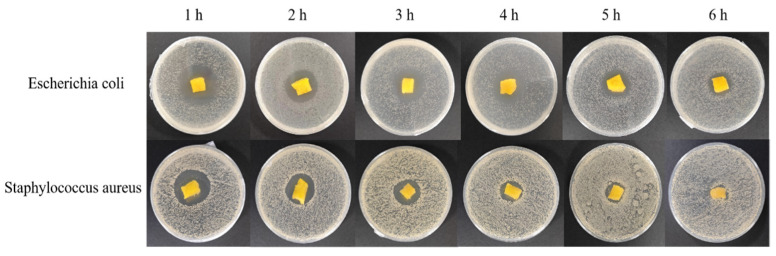
Photographs of the zones of inhibition after allowing the gelatin derivative to stand for different periods of time.

The inhibition rate of the bacteriostatic gelatin derivative for both *E. coli* and *S. aureus* gradually increased with increasing concentrations of GOD, reaching an inhibition rate of 99% when the concentration of the GOD solution reached 20,000 u/mL. The antibacterial gelatin derivative with 20,000 u/mL of GOD solution was tested for its antibacterial performance at different times (1, 2, 3, 4, 5, and 6 h) after fully absorbing 10 mg/mL of a glucose solution. The results are shown in Table 6 and Figure 10.

As shown in Table 6, with increasing exposure time, the diameter of the inhibition of the bacteriostatic gelatin derivatives for both *E. coli* and *S. aureus* gradually decreased, with values of 19.04 mm and 10.07 mm, respectively, after 6 h of exposure. The quantitative values of the bacteriostaticity of the antibacterial gelatin derivative with 20,000 u/mL of GOD solution were determined, and the results are shown (Table 7).

As shown in Table 7, the antibacterial gelatin derivative retained an inhibition rate > 99% even after 6 h. Therefore, the exposure time was extended to 12 and 24 h. The inhibition rates of the antibacterial gelatin derivatives for *E. coli* and *S. aureus* after 24 h were > 97%. Therefore, the efficacy range of the antibacterial gelatin derivative may be reduced if it is left on an open wound for more than 24 h.

## 4. Conclusions

In this study, a two-step enzymatic method involving protease-catalyzed hydrolysis and TGase-catalyzed cross-linking was used to prepare a dual-enzyme gelatin derivative. Compared with the TGase gelatin derivative, the dual-enzyme gelatin derivative exhibited higher swelling and water retention rates, as well as superior water vapor permeability. The dual-enzyme gelatin derivative had an improved swelling rate of 915.55%, a water retention rate of 43.89%, and a water vapor transmission rate of 486.72 g·m^−2^·24 h^−1^, which met the basic requirements of an ideal derivative. The average pore size of the dual-enzyme gelatin derivative was 150 µm, and its thermal stability was improved compared with that of the natural gelatin derivative. Glucose oxidase was cross-linked and immobilized on the dual-enzyme gelatin derivative using TGase, increasing the bacteriostatic rate (>99%) of the derivative for both *E. coli* and *S. aureus*. These findings indicate that dual-enzyme gelatin derivatives could be used as a novel hemostatic derivative for trauma treatment.

## Figures and Tables

**Table 1:** Orthogonal experimental design for alkaline protease action conditions.

Factors	Encodings	Level			
		1	2	3	4
Gelatin concentration (%)	A	6	8	10	12
Enzyme addition (u/mL)	B	5	10	15	20
Temperature (°C)	C	40	45	50	55
pH	D	6	7	8	9
Time (min)	E	2	4	6	8

**Table 2:** Orthogonal experimental design for TGase action conditions.

Factors	Encodings	Level		
		1	2	3
TG enzyme addition (u/mL)	A	50	100	150
Temperature (°C)	B	40	45	50
pH	C	6	7	8
Time (min)	D	30	60	90

**Table 3:** The degree of swelling and the rate of water vapor transmission for gels/wound dressings.

	Dressing Type	Numerical Value
Swelling rate	Starch/PVA/glycerin wound dressing [33]	114–473%
	Gelatin based fiber bio-mat [34]	456%
	Gelatin-based wound compress [35]	140%
	Alginate wound dressing [36]	5–15%
Water vapor transmittance	Gelatin-based wound dressing [35]	1.03 ± 0.05 mg cm^2^ h
	Hydroxypropyl methylcellulose succinate cross-linked chitosan hydrogel membrane [37]	708.70 g/m^2^/day
	Gelatin methylacrylyl nanofiber hydrogel [38]	15.8 mm s^−1^

**Table 4:** The diameter of the zone of inhibition produced by different concentrations of glucose oxidase after exposure to *Escherichia coli* and *Staphylococcus aureus*.

GOD Solution Concentration	5000 u/mL	10,000 u/mL	15,000 u/mL	20,000 u/mL
*Escherichia coli*	31.93 mm	34.09 mm	36.86 mm	39.47 mm
*Staphylococcus aureus*	30.42 mm	33.32 mm	33.76 mm	34.65 mm

**Table 5:** Bacteriostatic inhibition rates of the bacteriostatic gelatin derivatives with different concentrations of GOD.

GOD Solution Concentration	5000 u/mL	10,000 u/mL	15,000 u/mL	20,000 u/mL
*Escherichia coli*	90.6%	93.8%	96.4%	99.6%
*Staphylococcus aureus*	92.5%	96.1%	98.8%	99.1%

**Table 6:** Circle of inhibition diameters after different times of exposure to antibacterial gelatin derivative.

Time	1 h	2 h	3 h	4 h	5 h	6 h
*Escherichia coli*	34.08 mm	30.76 mm	29.81 mm	28.94 mm	24.11 mm	19.04 mm
*Staphylococcus aureus*	32.77 mm	22.44 mm	17.60 mm	16.42 mm	16.35 mm	10.07 mm

**Table 7:** The inhibition rate after exposing the bacteria to the antibacterial gelatin derivative with 20,000 u/mL of GOD solution for different periods of time.

Time	1 h	2 h	3 h	4 h	5 h	6 h	12 h	24 h
*Escherichia coli*	99.7%	99.5%	99.3%	99.6%	99.6%	99.3%	99.1%	98.6%
*Staphylococcus aureus*	99.5%	99.6%	99.5%	99.4%	99.5%	99.3%	99.0%	97.5%

## Data Availability

All generated and analyzed data used to support the findings of this study are included within the article.
